# Postural Control Strategies and Balance-Related Factors in Individuals with Traumatic Transtibial Amputations

**DOI:** 10.3390/s21217284

**Published:** 2021-11-01

**Authors:** Barbora Kolářová, Miroslav Janura, Zdeněk Svoboda, Petr Kolář, Dagmar Tečová, Milan Elfmark

**Affiliations:** 1 Kinesiology Laboratory, Department of Rehabilitation, University Hospital Olomouc, I.P. Pavlova 6, 779 00 Olomouc, Czech Republic; petr.kolar@fnol.cz; 2 Department of Clinical Rehabilitation, Faculty of Health Sciences, Palacký University Olomouc, Hněvotínská 976/3, 775 15 Olomouc, Czech Republic; d.tecova@seznam.cz; 3 Department of Natural Sciences in Kinanthropology, Faculty of Physical Culture, Palacký University Olomouc, třída Míru 117, 771 11 Olomouc, Czech Republic; miroslav.janura@upol.cz (M.J.); zdenek.svoboda@upol.cz (Z.S.); milan.elfmark@upol.cz (M.E.)

**Keywords:** transtibial amputation, postural control, posturography

## Abstract

Mechanisms behind compromised balance control in people with transtibial amputation need to be further explored, as currently little is known specifically about postural control strategies in people with traumatic transtibial amputation (tTTA). The aim of this study is to assess automatic and voluntary postural control strategies in individuals with unilateral tTTA compared to those in control subjects and to define the effect of balance-related factors on these strategies. Automatic posture reactions and volitional motion toward given direction using standardized posturographic protocols (NeuroCom) of the Motor Control Test (MCT) and Limits of Stability (LOS) were assessed in eighteen participants with tTTA and eighteen age-matched controls. Compared to the controls, the participants with tTTA bore less weight on the prosthetic leg (*p* < 0.001) during the MCT and had reduced inclination toward the prosthetic leg (*p* < 0.001) within the LOS. In the tTTA group, the weight-bearing symmetry and the inclination toward the prosthetic leg (*p* < 0.05) was positively correlated with prosthesis use duration (*p* < 0.05). The current study indicates that decreased utilization of the prosthetic leg in tTTAs represents adaptive postural control strategy, but as prosthesis use duration increased, the engagement of the prosthetic leg improved.

## 1. Introduction

Adequate postural control is necessary to effectively perform everyday activities and to prevent falls. Postural control is compromised in individuals after transtibial amputation (TTA), primarily due to the loss of sensory feedback and muscle control of the amputated leg and decreased range of motion of the prosthetic foot [1]. The ensuing active muscle power and body mass asymmetry, as well as the deteriorated somatosensory input, must be compensated for by altered balance control strategies [2,3]. One of the most obvious compensatory and adaptive postural control strategies in lower limb amputees is the decreased utilization of the leg with the prosthesis than that of the intact leg. After a TTA, most individuals rely automatically on their intact leg during standing [3,4,5,6] and walking [7,8]. This strategy is even more pronounced in situations with increased demands for postural control [3,5,9,10,11,12,13].

The decreased utilization of the prosthetic leg for posture control is compensated by altered biomechanics of the intact non-amputated limb, pelvis, and trunk [8,14]. This might increase the risk of developing pain symptoms [15] and secondary overuse musculoskeletal injury which has a relatively high incidence in lower limb amputees [16] and restricts their functional mobility [14,15]. From this perspective, the research focused on further understanding of underlying adaptive postural control mechanisms in TTA is desired [17,18] as maximizing the engagement of the prosthetic limb for postural control tasks after TTAs seems to play an important role in improving patient outcomes [19] and should be reflected during rehabilitation [2,3], prosthetic alignments, or arrangement of new prosthetic devices [18].

To maintain postural stability both automatic sensorimotor processing (beside others reflecting the adaptability and postural reactivity to unexpected environmental changes) and volitional processing have to be employed [1]. Factors, which have been previously suggested to have impact on automatic or volitional postural control in individuals after lower limb amputation include etiology of amputation [3,4], age [4], activity level, duration of prosthesis use [5,20], stump length [21,22], prosthetic components and alignment [23], fall history [3,10] or fear of falling [24]. However, there is little evidence how these factors may influence the engagement of the prosthetic and/or intact leg with respect to both automatic and volitional aspects of postural control in TTAs.

Postural control mechanisms may be measured using computerized dynamic posturography [14,15]. Multiple studies for lower limb amputees used the standardized posturographic protocols of the motor control test (MCT) for the evaluation of automatic postural responses to external perturbations, and the limits of stability test (LOS) for the assessment of the maximum voluntary body inclination without changing the base of support. These tests proved to be highly reliable [10,11,19,24,25]. Despite the fact that the etiology of amputation seems to play crucial role in balance control [26], there are only a limited number of studies evaluating postural control strategies in these patients and even more specifically, in traumatic TTA.

The main aim of the present study is to assess the automatic and voluntary postural control strategies in individuals after traumatic TTAs and compare them with control subjects—to define the relevant parameters of impaired postural control in which TTAs due to trauma differ from healthy subjects. Partial aim was to define the impact of the balance-related factors such as prosthesis use duration, stump length, and fall history on these strategies. The main hypothesis was that the TTA group results would differ from the control group when performing automatic and volitional postural control tasks with regard to decreased utilization of their prosthetic lower limb.

## 2. Materials and Methods

### 2.1. Participants

Eighteen individuals with traumatic TTAs were recruited from the regional prosthetic centers and control subjects were recruited by announcement at a local university. Inclusion criteria for participation in the experimental TTA group were: (1) age 18–70 years, (2) underwent transtibial amputation due to trauma, (3) at least 6 months of daily use of an energy-storage and release prosthetic foot that was properly fitted by a trained and certified prosthetist, and (4) independent mobility outside the home (K-level 3 or 4). Exclusion criteria were self-reported fear of falling during daily activities, including standing or walking, and presence of orthopedic, neurological, or cognitive disorder. The eighteen age- and sex-matched healthy controls were selected using the same exclusion criteria, including the absence of any orthopedic, neurological, or cognitive disorder, the same as in the experimental group. None of the participants reported experiencing pain during the testing. In the experimental group, seven participants were classified as fallers with at least one fall during the year prior to testing [9], while the other eleven were non-fallers. In the control group, fallers were not identified. The local ethics committee approved the study and informed consent was obtained from all the subjects involved in the study.

### 2.2. Experimental Protocol

The computerized dynamic posturography (Smart EquiTest System, NeuroCom International Inc., Clackamas, OR, USA) was performed with a dual moveable force plate with a sampling frequency of 100 Hz. The platform consisted of two force plates (23 × 46 cm each) connected by a pin joint, with four vertically oriented corner transducers to measure the vertical forces applied to the force plate, and one horizontally oriented transducer to measure the shear forces in the forwards-backwards direction parallel to the floor [9,23,27]. Total vertical force, which is calculated as a sum of the vertical forces measured by the four corner transducers, was used to determine the subjects’ weight. The subjects’ weight and height entered by the operator into the system were used to derive the center of gravity (COG) based on a computer model of body dynamics [28].

The same methods were used to evaluate aspects of automatic and voluntary postural control strategies in both the experimental and control groups. Both groups wore shoes during testing. The standardized MCT and LOS test protocols were performed with all participants according to the manufacturer’s instructions [27] as described in previous studies [9,10,17,21,22,23,29]. In cases of a mistrial (e.g., caused by foot displacement during testing), the trial was repeated.

### 2.3. Outcome Measurements

#### 2.3.1. Motor Control Test

The MCT was used to evaluate the automatic postural reactions in response to the support surface translations. During the test, the force plate translates backward and forward horizontally in three speeds (slow, medium, and large). The platform speed is normalized by the subject’s height to produce an estimated COG sway angle disturbance of 2.8 deg/s (slow), 6 deg/s (medium), and 8 deg/s (large). Every translation is performed in triplicates and the results from the three repetitions are averaged to obtain the final scores. For the MCT, weight symmetry and latency were measured and evaluated. **Weight bearing symmetry** score (WBS, %) was the percentage of total body weight borne by each leg during the automatic postural response, defined as weight-bearing of the prosthetic leg/weight-bearing of the non-amputated leg (TTA group) × 100 or weight-bearing of the right leg/weight-bearing of the left leg (control group) × 100. A score of 100 meant that the weight was borne equally by both legs, while a score lower than 100 indicated that more weight was borne by the left leg in the control group and by the non-amputated leg in the TTA group. **Latency** (ms) was the time in milliseconds between the onset of the translation movement during the MCT and the onset of the patient’s active force response to the support surface movement (shear forces, which are a result of the body’s COG acceleration when the platform moves). Latency was calculated separately for each leg. However, substantial unloading of one leg during testing may influence the ability to generate an active force response and thus alter the latency calculation [27]. So, the latency score for the prosthetic leg was not detectable in most cases and for this reason this parameter was not evaluated in the TTA group, similarly as in previous published studies [10,11]. The latency score was evaluated in control subjects for both legs.

#### 2.3.2. Limits of Stability

The LOS test evaluated the voluntary ability to shift the COG from a central position out towards one of eight targets in straight or diagonal directions in the order as follows: forward, forward-right, right, backward-right, posterior, posterior-left, left, and anterior-left. The targets reflect a theoretical LOS of 100% [26]. In the test, the participants used visual feedback to achieve the most precise stability limit through body inclination toward the target direction. The sequence of targets was presented in a standardized clockwise direction. All participants performed practice trials in all directions prior to starting the test for data collection [21]. Within the LOS test, four outcomes were measured and evaluated for each target direction. The **maximum excursion** (MXE, %) was the maximal COG distance toward the target. The **endpoint excursion** (EPE, %) was the COG distance reached by the participants at the initial attempt toward the target. The **movement velocity** (MVL, deg/s) was the average speed of the COG movement. Finally, the **direction control** (DCL, %) was depicted as a percentage of the total on-axis shift of the COG toward the target (a value of 100% indicated a completely on-axis movement) and reflected the smoothness of the COG movement. For MXE and EPE, the measure was described as a percentage of a theoretical LOS of 100%, where 100% indicates better stability.

### 2.4. Statistical Analyses

A sample size was estimated using two-way ANOVA with factors group and limb. When we consider significance level α = 0.05, power = 0.80 and at least medium effect size = 0.25, the resulting total number of participants is 34 (17 participants in each group). Descriptive statistics (means ± standard deviations) were used to describe all relevant demographic and measured variables. The data distribution was tested by the Kolmogorov–Smirnov test for normality. Independent *t*-tests were used to compare the differences in demographics (age, weight, and height) between the control and experimental groups.

To test the first hypothesis, two-way ANOVA and independent *t*-tests were used for comparisons between the MCT and LOS test outcomes for the control and experimental groups to define the relevant parameters of impaired postural control in traumatic TTAs, while effect size was measured using *eta squared* (η^2^ ≥ 0.01 indicated a small effect, η^2^ ≥ 0.06 a medium effect, and η^2^ ≥ 0.14 a large effect). To avoid type I error, post-hoc multivariate analyses were performed using Bonferroni’s correction for MCT values of *p*  ≤  0.0083 (0.05/6) and LOS values of *p*  ≤  0.0063 (0.05/8). For each posturographic outcome, Cohen’s effect size was calculated (*d*  ≥ 0.20 indicated a small effect, *d*  ≥  0.50 a moderate effect, and *d*  ≥  0.80 a large effect). To test the second hypothesis, bivariate associations between TTA group characteristics (prosthesis use duration and stump length) and relevant posturographic outcomes were calculated. Since the participant characteristics were not normally distributed, non-parametric Spearman’s correlation coefficients (*r*) were used for further analyses. The Mann–Whitney *U*-test was used to compare the outcomes of the MCT and LOS test for TTA group fallers and non-fallers. Statistical analyses were performed using Statistica v. 12.0 (StatSoft, Inc., Tulsa, OK, USA).

## 3. Results

The participant characteristics are shown in Table 1. No significant inter-group differences in age, height, or weight were found.

In the MCT, the WBS was significantly lower in the individuals after amputation than in the control group (*p* < 0.001; η^2^ = 0.19). Post-hoc comparisons revealed that the TTA group performed weight-bearing asymmetrically during the MCT with preference given to their non-amputated leg for small and medium backward translations and all forward translations. For the latency score, no significant differences were found. For the results of MCT see Table 2.

For the LOS test, the multivariate analysis results revealed an overall significant difference between the control and experimental groups in the parameters of MXE (*p* < 0.001, η^2^ = 0.16), EPE (*p*  <  0.001, η^2^ = 0.06), and MVL (*p*  <  0.001, η^2^ = 0.14). When each LOS parameter was considered, the inter-group difference remained significant for MXE and EPE for inclination to the amputated side and diagonally forward amputated side, as well as for MXE in the forward direction or backward diagonal direction to the amputated side. For MVL, a significant difference was found in the inclination in the backward diagonal direction toward the non-amputated leg. For DCL, no significant inter-group difference was found. For the results of LOS see Table 3.

Bivariate associations between posturographic outcomes and TTA group characteristics are presented in Table 4 for the MCT and in Table 5 for the LOS test. The stump length and prosthesis use duration negatively correlated with the weight bearing score for small and large forward translations within the MCT (*r* < −0.5, *p* < 0.05) and positively corelated with the MXE and EPE score toward the prosthetic side within the LOS test (*r >* 0.5, *p*  <  0.05). However, history of fall in previous year in the TTA group was not associated with any of tested posturographic parameters.

## 4. Discussion

### 4.1. Aspects of Automatic and Volitional Postural Control Strategies

During forward translations in the MCT, the TTA group had a significant weight-bearing asymmetry with a preference for their non-amputated leg, which is consistent with results of studies using the similar testing protocol with TTA groups of non-fallers [9], or in groups with TTAs due to trauma [10]. In the present study, the latencies of postural reactions realized via the non-amputated leg during the MCT were comparable to those in the control group. Therefore, we posit that the sensory processing to evoke an efficient reaction for the intact limb might remain unchanged for individuals in the TTA group. Molina-Rueda et al. [10] found shorter latencies for medium backward and forward translations in individuals with traumatic TTAs in their non-amputated limb in comparison to individuals with vascular TTAs. From these and from the results of the present study, the increased reliance on the non-amputated leg might be considered as an effective compensation strategy as suggested previously [17]. Particularly in the patients with traumatic TTA, where the intact leg provides an appropriate immediate proprioceptive motor feedback, we see the ability to react effectively to unexpected environmental changes to prevent falls, as opposed to, for example, in those with divascular etiology of amputation. Vascular disease is often manifested as a sensory deficit in both legs [30] and is a risk factor for falls [31]. However, the weight bearing asymmetry in lower limb amputees might be associated with their impaired balance [3].

During the LOS test, the TTA group had a significantly decreased maximal voluntary inclination on the first attempt to reach this maximum toward the prosthetic side and toward the prosthetic side diagonally compared to the control group. The decreased LOS in the TTA group toward the amputated side has been reported earlier for the first attempt to reach the stability limits (parameter EPE) [19,20,23], even in studies which included subjects after amputation due to both vascular and non-vascular etiology [20,23]. Reduced LOS in direction of prosthetic leg most probably result from deficit of sensorimotor control and lead to decreased functional base of support [20,23,32]. Our results reflect the decreased ability to shift the body in the direction of prosthetic leg and thus might limit daily living activities as walking or reaching for objects and increase the risk of falling [32].

According to this part of our study results, we confirm here, that specifically in individuals with traumatic TTAs, the prosthetic leg tends to be utilized less for both automatic and volitional postural tasks than the non-amputated leg. This strategy reflects the actual postural control mechanisms after TTAs [4], and might result in impaired balance control or secondary overuse musculoskeletal injury. Thus, the question remains, which factors contribute to enhancing the utilization of the amputated leg and improving automatic and volitional aspects of postural control specifically in traumatic TTAs.

### 4.2. The Influence of Balance-Related Factors on Postural Control Mechanisms

The inter-group comparison of the TTA and control groups highlight the relevant parameters of postural control strategies that were significantly impaired in the TTA group. As we hypothesized, stump length and prosthesis use duration were related to these outcomes. Specifically, we found some statistically significant, moderate positive correlations between prosthesis use duration and relevant LOS and MCT outcomes, and negative correlations between the stump length and relevant MCT outcomes.

Our results suggest that prosthesis use duration considerably contributes to the improved utilization of the prosthetic leg for automatic and voluntary postural control mechanisms. For example, the longer the prosthesis use, the better the weight-bearing symmetry within automatic reactions during platform translation in a forward direction. In addition, there was an improved conscious inclination toward the prosthetic side. Improved symmetrical limb loading was reported in a study on experienced versus first-fitted unilateral prosthesis users [5] and another study on short-term transtibial prosthesis users [20]. However, our results indicate that engagement of the prosthetic leg for both automatic and voluntary postural tasks may improve for long-term transtibial prosthesis users. This effect might be a reflection of the increased confidence of amputees to load the prosthetic leg [6] as a result of adaptive mechanisms such as central reorganization processes, which continue to progress over the lifespan as experience and practice increase [33]. These findings highlight the importance of daily prosthesis use and the importance of rehabilitation in individuals after TTA, even in experienced prosthesis users.

Our results also suggest that individuals with a longer stump length perform more severe asymmetrical weight-bearing (with a preference for the intact leg) in forward directions during the MCT. These results were surprising as previous studies have suggested that a longer stump improves standing stability [21,22], walking parameters [34], and functional mobility [35]. In TTAs the ideal residual tibia length ranges from 12.5 to 17.5 cm [36]. The residual limb length in the present study ranged from 12 to 35 cm. Therefore, the results probably reflect a maladaptive impact of an excessively long stump on postural control mechanisms and should be further explored.

We found no influence of fall history on the evaluated postural strategies in our study. Previous studies have suggested that individuals with TTAs defined as fallers have no limb preference for standing during platform translation within the MCT [8] and that the increased fear of falling is associated with a decreased excursion in the backward direction measured in the LOS test [24]. Since we found asymmetrical weight-bearing with intact limb preference during the MCT but no decreased performance during the LOS test in the backward direction, we deduce that the traumatic TTA group tested in our study, who all were fully independent walkers, had sufficient confidence for the test situation regardless of having a fall history in the previous year.

### 4.3. Study Limits

The results of this study are limited to adults after traumatic TTA with a relatively high activity level in one geographic region. Potential sampling bias was introduced as all participants were asked to volunteer for the study, rather than being randomly selected, and thus the obtained data may not be representative of the general population for the experimental and control groups. The residual confounders for this study that went unexamined were the absence of pain measurement, as prosthetic limb pain and low back pain are common in lower limb amputees and can restrict their functional mobility [15]. The association between the engagement of the prosthetic limb for postural control tasks and the presence of pain might be useful for future research on prosthetic rehabilitation management as the maximum possible involvement of the prosthetic leg for postural control is required to diminish the postural asymmetries and to minimize musculoskeletal disorders.

## 5. Conclusions

Our study results confirmed the preference of non-amputated leg specifically in subjects after transtibial amputation due to trauma as an adaptive balance control mechanism for both automatic and voluntary postural tasks. Within the assessed posturographic tests, the TTA group performed asymmetrical lower limb loading to favor the non-amputated leg during automatically elicited postural reactions and displayed a decreased ability to voluntarily shift their body in the direction of the prosthetic limb. Based on the study results we further conclude that improved engagement of the prosthetic leg for automatic and voluntary postural tasks was positively associated with the prosthesis use duration but not with the stump length or the history of fall in our tested group.

## Figures and Tables

**Table 1:** Participant characteristics.

Control Group				Traumatic Transtibial Amputees Group							
Gender	Age (years)	Height (cm)	Weight (kg)	Gender	Age (years)	Height (cm)	Weight (kg)	Amputated Leg	Prosthesis Use (years)	Stump Length (cm) ^1^	At Least One Fall in the Last Year
F	23	164	63	F	23	165	67	left	1	28	Yes
M	26	183	81	M	25	198	87	left	2	27	No
M	36	169	76	M	36	185	82	left	11	19	No
M	42	182	73	M	40	192	85	right	7	17	Yes
M	44	189	94	M	41	177	101	left	9	20	No
M	46	171	80	M	41	197	78	right	4	31.5	No
M	48	177	67	M	46	180	88	left	0.8	14	No
F	48	170	68	F	49	170	105	right	4	17.5	Yes
M	49	172	76	M	49	181	85	right	3	22	Yes
M	50	182	117	M	52	184	74	left	2	17	No
M	52	188	85	M	52	176	79	left	21	18.5	Yes
M	56	178	88	M	54	170	85	left	5	25	No
M	57	176	84	M	54	172	89	left	23	21	No
F	57	172	66	F	54	160	90	right	0.7	12	Yes
M	59	177	84	M	58	175	106	right	0.8	17	No
M	59	179	74	M	63	172	76	right	15	35	No
M	62	185	85	M	69	180	115	left	3	21	No
M	67	173	75	M	69	172	95	left	45	12	Yes
Mean	48.94	177.6	79.78		48.61	178.11	88.17		8.74	20.81	
SD	11.73	6.88	12.49		12.85	10.29	12.34		11.33	6.37	

^1^ Measured as the distance between the lateral femorotibial inter-condylar line and the stump tip, M—male, F—female.

**Table 2:** Motor control test results.

Translation Type	Control Group	TTA Group	*p*	*d*	Translation Type	Control Group	TTA Group	*p*	*d*
Weight bearing symmetry score (%)									
Small_B	98.17 (6.74)	87.33 (12.12)	0.003	1.10	Small_F	96.28 (7.43)	86.22 (12.58)	0.006	0.97
Medium_B	97.11 (5.93)	87.11 (12.87)	0.006	1.00	Medium_F	91.10 (21.06)	81.50 (21.14)	0.007	0.96
Large_B	95.67 (5.67)	83.56 (20.47)	0.025	0.81	Large_F	96.61 (6.41)	85.56 (12.65)	0.002	1.1
**Latency score (ms) ^1^**									
Small_B	147.22 (15.65)	154.44 (17.90)	0.210	0.43	Small_F	151.67 (22.03)	151.11 (20.26)	0.940	0.03
Medium_B	141.11 (12.78)	142.78 (11.79)	0.690	0.14	Medium_F	145.56 (15.80)	148.89 (27.42)	0.660	0.15
Large_B	139.44 (13.92)	135.56 (13.38)	0.400	0.28	Large_F	140.56 (13.49)	133 (11.88)	0.097	0.57

TTA—transtibial amputees, B—backward, F—forward, *d*—Cohen’s *d*. ^1^ Latency scores are for the right leg in the control group and for the non-amputated leg in the TTA group.

**Table 3:** Limits of Stability test results.

Direction	Control Group	TTA Group	*p*	*d*	Direction	Control Group	TTA Group	*p*	*d*
Maximum excursion (%)									
Forward	87.61 (12.56)	72.28 (14.25)	0.002	1.14	Backward	77.39 (10.58)	74.56 (17.98)	0.568	0.19
Forward_I/R	87.44 (14.32)	81.67 (12.03)	0.199	0.44	Backward_P/L	93.22 (9.92)	80.56 (12.97)	0.002	1.1
I/Rt	88.00 (10.82)	80.06 (8.64)	0.020	0.81	P/L	91.11 (7.78)	76.00 (7.49)	<0.001	1.98
Backward_I/R	93.78 (10.20)	86.78 (13.09)	0.082	0.60	Forward_P/L	94.78 (11.95)	71.06 (17.21)	<0.001	1.60
**Endpoint excursion (%)**									
Forward	75.56 (19.16)	62.72 (17.05)	0.041	0.71	Backward	56.11 (13.67)	49.89 (18.08)	0.252	0.39
Forward_I/R	76.11 (16.72)	75.89 (12.47)	0.964	0.57	Backward_P/L	77.22 (14.82)	66.72 (21.60)	0.098	0.57
I/R	79.00 (8.85)	70.94 (18.08)	0.102	0.02	P/L	81.44 (7.98)	66.33 (12.52)	<0.001	1.44
Backward_I/R	63.11 (19.04)	77.5 (16.68)	0.405	0.28	Forward_P/L	82.61 (24.11)	62.22 (18.15)	0.007	0.96
**Movement velocity (deg/s)**									
Forward	4.72 (1.91)	4.00 (2.52)	0.064	0.40	Backward	3.67 (1.27)	2.56 (1.10)	0.008	0.93
Forward_I/R	5.83 (2.1)	4.42 (1.99)	0.047	0.69	Backward_P/L	5.21 (2.07)	4.04 (2.18)	0.109	0.55
I/R	5.82 (2.76)	4.47 (2.16)	0.088	0.59	P/L	6.49 (2.91)	4.99 (2.54)	0.026	0.55
Backward_I/R	5.89 (2.08)	3.37 (1.47)	<0.001	1.40	Forward_P/L	5.93 (1.9)	3.99 (2.52)	0.013	0.87
**Directional control (%)**									
Forward	88.22 (5.11)	82.22 (12.54)	0.073	0.63	Backward	75.78 (9.97)	67.89 (15.98)	0.085	0.59
Forward_I/R	75.28 (10.28)	78.50 (10.34)	0.355	-0.31	Backward_P/L	64.17 (23.19)	64.11 (16.06)	0.993	0.00
I/R	84.83 (6.71)	84.28 (8.71)	0.831	0.07	P/L	84.56 (7.28)	86.89 (4.60)	0.258	−0.38
Backward_I/R	93.78 (10.20)	74.17 (12.20)	0.046	-0.69	Forward_P/L	79.44 (7.96)	77.44 (12.67)	0.574	0.19

P—prosthetic leg in TTA group, R—right leg in control group, I—intact leg in TTA group, L—left leg in control group, *d*—Cohen’s *d*.

**Table 4:** Associations between amputation characteristics and outcomes of motor control test.

Translation Type	Stump Length		Prosthesis Use Duration		Translation Type	Stump Length		Prosthesis Use Duration	
	*r*	*p*	*r*	*p*		*r*	*p*	*r*	*p*
Weight bearing symmetry score (%)									
**Small_B**	−0.19	0.45	0.37	0.13	Small_F	**−0.53**	**0.02**	**0.47**	**0.048**
Medium_B	−0.26	0.29	0.21	0.41	Medium_F	−0.44	0.08	0.27	0.29
Large_B	−0.19	0.45	0.3	0.24	Large_F	**−** **0.55**	**0.02**	**0.52**	**0.03**
**Latency score (ms)**									
Small_B	−0.25	0.32	0.43	0.07	Small_F	−0.13	0.62	0.12	0.63
Medium_B	**−0.56**	**0.02**	0.32	0.20	Medium_F	−0.45	0.06	**0.62**	**0.01**
Large_B	−0.38	0.12	0.34	0.17	Large_F	**−** **0.54**	**0.03**	0.20	0.44

B—backward, F—forward, *r*—correlation coefficient.

**Table 5:** Associations between amputation characteristics and outcomes of limits of stability test.

Direction	Stump Length		Prosthesis Use Duration		Direction	Stump Length		Prosthesis Use Duration	
	*r*	*p*	*r*	*p*		*r*	*p*	*r*	*p*
Maximum excursion (%)									
Forward	−0.18	0.47	0.16	0.52	Backward	0.36	0.14	−0.23	0.36
Forward_I/R	−0.40	0.10	0.34	0.17	Backward_P/L	−0.10	0.69	−0.01	0.97
Intact/Right	−0.01	0.95	0.24	0.34	Prosthetic/Left	0.03	0.92	**0.54**	**0.02**
Backward_I/R	−0.29	0.24	−0.01	0.98	Forward_P/L	−0.07	0.77	0.21	0.41
**Endpoint excursion (%)**									
Forward	−0.33	0.19	0.27	0.27	Backward	0.45	0.06	−0.3	0.23
Forward_I/R	−0.25	0.32	0.19	0.44	Backward_P/L	−0.26	0.30	−0.14	0.58
Intact/Right	−0.16	0.54	0.03	0.92	Prosthetic/Left	−0.28	0.27	**0.63**	**<0.001**
Backward_I/R	−0.36	0.15	0.26	0.29	Forward_P/L	−0.21	0.40	0.20	0.42
**Movement velocity (deg/s)**									
Forward	−0.13	0.62	0.29	0.24	Backward	0.30	0.23	−0.08	0.74
Forward_I/R	−0.02	0.95	0.21	0.41	Backward_P/L	−0.28	0.27	0.19	0.45
Intact/Right	−0.13	0.62	0.13	0.61	Prosthetic/Left	−0.11	0.66	0.14	0.58
Backward_I/R	0.03	0.91	0.05	0.86	Forward_P/L	0.20	0.24	−0.21	0.40
**Directional control (%)**									
Forward	−0.02	0.92	−0.11	0.67	Backward	0.16	0.53	−0.25	0.31
Forward_I/R	−0.19	0.44	−0.14	0.57	Backward_P/L	0.35	0.15	−0.14	0.57
Intact/Right	0.43	0.08	−0.14	0.59	Prosthetic/Left	−0.01	0.96	0.42	0.09
Backward_I/R	0.42	0.09	−0.262	0.29	Forward_P/L	−0.08	0.75	0.01	0.96

P—prosthetic leg in TTA group, R—right leg in control group, I—intact leg in TTA group, L—left leg in control group, *r*—correlation coefficient.

## Data Availability

The data presented in this study are available on request from the corresponding author.
